# Host Barriers to In Vivo HSC Gene Therapy with Helper-Dependent Adenovirus Vectors

**DOI:** 10.3390/v18101078

**Published:** 2026-09-30

**Authors:** Anna K. Anderson, André Lieber, Chang Li

**Affiliations:** 1 Department of Medicine, University of Washington, Seattle, WA 98195, USAlieber00@uw.edu (A.L.); 2 Department of Pathology and Laboratory Medicine, University of Washington, Seattle, WA 98195, USA; 3 State Key Laboratory of Virology and Biosafety, Wuhan Institute of Virology, Chinese Academy of Sciences, Wuhan 430071, China

**Keywords:** gene therapy, hematopoietic stem cells, helper-dependent adenovirus, bone marrow, immune response, vector engineering

## Abstract

Ex vivo hematopoietic stem cell (HSC) gene therapy strategies have proved effective for the treatment of various diseases, with recent advances leading to the clinical approval of several cell products. To overcome issues associated with the cost and technical complexity of ex vivo strategies, we turn towards in vivo strategies that will allow us to avoid myeloablative transplantation and broaden access. Advances in the engineering of adenoviruses have made them a promising platform for the targeted in vivo delivery of gene therapy vectors to HSCs. This narrative review highlights host barriers to in vivo HSC gene therapy using helper-dependent adenovirus vectors including innate and adaptive immune responses in the blood, virus uptake and sequestration by non-target cells and tissues, difficulty accessing the bone marrow niche, and the inefficient targeting of quiescent HSCs. We present recent advances aimed at overcoming these barriers including immune modulation and vector engineering strategies, improved predictive models, novel HSC targeting strategies, and enrichment of modified HSCs. This forward-looking review highlights a path towards achieving the clinical translation of predictable, safe and accessible in vivo adenovirus-mediated HSC gene therapies.

## 1. Introduction

Recent clinical successes have established hematopoietic stem cell (HSC) gene therapy as a promising treatment for a growing number of inherited and acquired disorders affecting the blood and immune system. Owing to their capacity for long-term self-renewal and multilineage differentiation, HSCs represent an ideal target for durable genetic modification, enabling lifelong production of genetically corrected blood cells. Ex vivo HSC gene therapies, which involve the myeloablative transplantation of genetically modified HSCs, have recently gained FDA approval for the treatment of multiple diseases [1]. While these advancements have demonstrated the clinical efficacy of HSC-based gene therapy, their accessibility remains limited by high cost and the need for complex procedures. This has driven increasing interest in the development of in vivo HSC gene therapy strategies, which have the potential to expand access through reduced cost and technical complexity.

In vivo modification of HSCs requires the targeted delivery of transgenes to long-term repopulating HSCs within the bone marrow. Among the delivery platforms explored for this purpose, adenoviral vectors show unique promise. They are among the most widely studied gene delivery systems, with ClinicalTrials.gov listing more than 650 human clinical trials employing these vectors, predominantly in the areas of cancer therapy and vaccination. Adenoviral vectors possess several characteristics that make them particularly attractive for clinical gene therapy applications, including their large packaging capacity, high transduction efficiency, scalable manufacturing capability, and ability to accommodate complex gene editing cargos. In addition, adenoviral vectors generally remain episomal unless specifically formulated to mediate genomic integration, thereby minimizing the risk of insertional mutagenesis.

Progressive deletion of adenoviral coding regions has resulted in successive generations of vectors with reduced viral gene expression and increased transgene carrying capacity. First-generation vectors lack the E1 region, rendering them replication deficient, and the E3 region, providing additional space for transgene insertion. Second-generation vectors retain the E1 and E3 deletions while incorporating additional deletions within the E2 and/or E4 regions, further reducing viral gene expression. Third-generation helper-dependent (“gutless”) adenoviral vectors (HDAds) contain no viral coding sequences and therefore do not express viral proteins, making them substantially less immunogenic than earlier generation vectors while providing the greatest cloning capacity. HDAds are replication deficient and are termed “helper-dependent” because their production relies on coinfection of a specialized producer cell line with a helper virus that supplies the viral proteins *in trans* required for vector assembly. More than 50 human adenovirus serotypes have been identified and are classified into species A–G. While first-generation vectors have been developed from both species B and species C adenoviruses, current HDAd platforms are primarily based on species C Ad5 and Ad6. Production of species B HDAds remains relatively limited due to unknown localization of their packaging signals.

The use of HDAds is particularly important for gene therapies targeting HSCs. Unlike first-generation vectors, HDAds do not express early adenoviral proteins that can impair HSC differentiation and long-term engraftment. Current HSC-targeted HDAd vectors are based on Ad5 or Ad6 capsids engineered to recognize receptors expressed on primitive HSCs through replacement of the native fiber knob with that of group B adenoviruses. Our laboratories currently employ four principal vectors: HDAd5/35++, HDAd6/35++, HDAd5/3+, and HDAd6/3+, which target either the CD46 or desmoglein 2 (DSG2) receptors expressed on HSCs rather than the coxsackievirus and adenovirus receptor (CAR) used by most adenoviral serotypes. These receptor-targeting strategies have enabled efficient in vivo HSC transduction in multiple preclinical animal models [2,3,4]. In addition, HDAd vectors have now entered clinical evaluation, with two ongoing clinical trials investigating helper-dependent adenoviral vectors for the treatment of HER2-positive solid tumors (NCT03740256) and X-linked chronic granulomatous disease (X-CGD) (NCT06876363) [5].

As HDAd-mediated HSC gene therapy advances toward clinical translation, a comprehensive understanding of the biological barriers encountered following systemic vector administration has become increasingly important. Although much of our current understanding of adenovirus-host interactions has been derived from studies using first-generation Ad5 vectors, helper-dependent and first-generation adenoviral vectors share similar mechanical properties and transduction mechanisms. Consequently, many of the fundamental interactions between adenoviruses and blood components, tissues, and the immune system remain directly applicable to HDAds [6]. In this narrative review, we examine the host barriers that limit the safety and efficacy of adenoviral vector-mediated in vivo HSC gene therapy and discuss emerging engineering and therapeutic strategies aimed at overcoming these challenges to enable the safe and effective clinical translation of HDAd-mediated HSC gene therapy. Literature was selected based on the authors’ research experience and expertise, and was identified through targeted searches of PubMed using key words related to the relevant topics, supplemented by manual screening.

## 2. Barriers

### 2.1. Bone Marrow

#### 2.1.1. Niche Access

**Figure 1:**
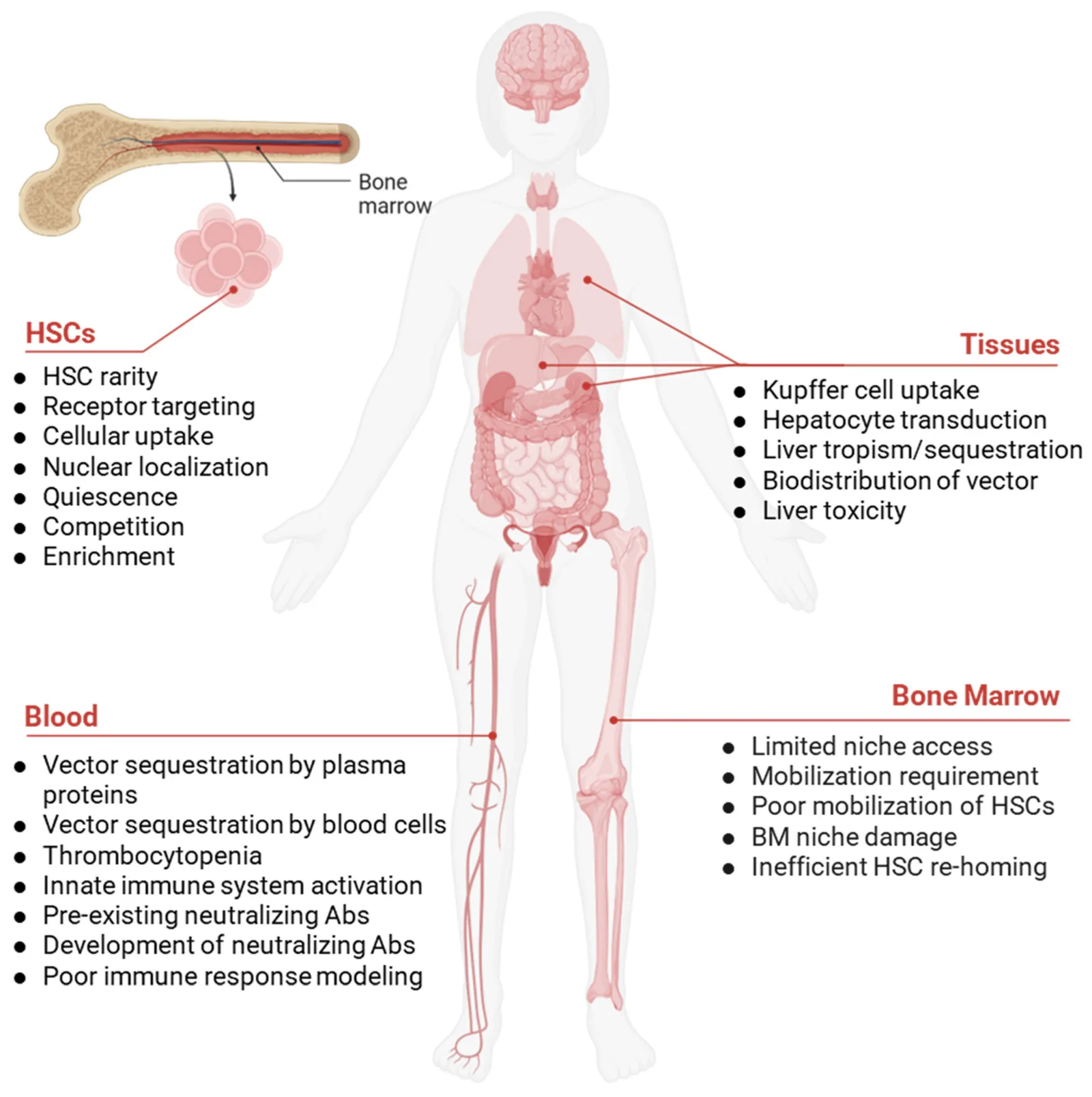
Host barriers to in vivo HSC gene therapy. HSCs, hematopoietic stem cells; BM, bone marrow; Abs, antibodies. Created in BioRender. Anna K. Anderson. (2026) https://app.biorender.com/illustrations/6a18840c1cc9c4a8c010be8c.

Hematopoietic stem cells (HSCs) reside within a specialized niche in the bone marrow, where they interact with non-hematopoietic cells including mesenchymal stem cells, osteoblasts, vascular endothelial cells, and megakaryocytes. These interactions regulate the proliferation, self-renewal, differentiation and localization of HSCs and contribute to the maintenance of phenotypically and functionally distinct subpopulations of HSCs within the marrow [7,8]. HSCs typically exist in a dynamic equilibrium between a quiescent non-dividing state and an activated state in which they proliferate and mature into multipotent progenitors [9]. For in vivo HSC gene therapy to achieve durable therapeutic efficacy, long-term repopulating HSCs must be genetically modified without compromising their capacity for self-renewal and multilineage differentiation. The targeting of HSCs within the bone marrow by systemically administered adenoviral vectors is constrained by several physical and biological barriers, including the extracellular matrix and vascular endothelium (Figure 1). Following intravenous administration, vectors must traverse the tightly regulated sinusoidal endothelium that separates the bone marrow vasculature from the hematopoietic niche. HSC trafficking across this barrier is an active and tightly regulated process governed primarily by chemokine signaling through the CXCR4-CXCL12 axis and by adhesion interactions involving α4β1 integrin and vascular cell adhesion molecule 1 (VCAM-1) [10]. Adenoviral vectors do not engage these physiological trafficking pathways and must therefore rely predominantly on vascular permeability and passive transport to across the endothelium and penetrate the surrounding stroma [11]. Bone marrow sinusoids have a discontinuous basement membrane and endothelium and are considered permeable. This said, imaging suggests that transport across the sinusoidal endothelium is size dependent and limited by pore dimensions, with permeability decreasing progressively as particle diameter increases from 4 nm to 32 nm [12]. Adenoviruses range from 90–100 nm in diameter, meaning that their size and structure likely limits passive diffusion through the endothelium and thereby restricts access to niche-resident HSCs. Intraosseous injection of viral vectors has been explored to bypass this barrier and to allow HSCs to be retained in their native microenvironment [13,14]. However, in large animal models, intraosseous delivery remains invasive and is limited by the requirement for multiple injection sites and inefficient vector diffusion within the bone marrow cavity, which restricts access to HSCs localized within endosteal niches [15]. Recognizing this barrier, recent advances have focused on enhancing HSC accessibility through transient niche modulation and mobilization.

#### 2.1.2. Mobilization

Mobilization refers to the induction of HSC egress from the bone marrow into the peripheral blood by mobilizing agents. These agents disrupt molecular interactions that retain HSCs within their niche, promoting detachment from the bone marrow stroma and/or egress into the bloodstream. Molecular targets for mobilization agents typically fall within either the CXCR4/CXCL12 or VLA-4/VCAM-1 axes. In a clinical setting, the most commonly used mobilization drug is granulocyte colony stimulating factor (G-CSF) which promotes the differentiation and division of granulocytes and activates the release of proteases, leading to the cleavage of adhesion molecules which tether HSCs within their niche [16,17]. In the context of in vivo HSC gene therapy, mobilization increases the accessibility of circulating HSCs and has been shown to enable more efficient transduction by intravenously delivered vectors [18,19,20]. Despite this advantage, conventional mobilization presents several challenges. First, around 10–20% of patients are considered “poor responders” and fail to achieve efficient mobilization with the use of common agents such as G-CSF. Additionally, conventional mobilization regimens are nonspecific to HSCs. The concurrent mobilization of committed leukocytes leads to vector sequestration and inflammatory cytokine release [21]. Finally, mobilization with G-CSF may pose risks for patients with certain conditions. In patients with β-thalassemia or sickle cell disease (SCD), diseases for which in vivo HSC gene therapy is being explored, G-CSF-mediated mobilization may cause severe or potentially life-threatening complications [22]. Recently, efforts have been focused on finding alternative mobilization agents to overcome these barriers. Plerixafor (AMD3100) is a clinically approved CXCR4 inhibitor that reduces HSC binding to the bone marrow stroma which has been shown to be effective in combination with lower G-CSF doses in patients with β-thalassemia, and to produce more selective mobilization of HSCs [20,23,24]. Motixafortide is another clinically approved CXCR4 inhibitor found to be effective in patients with SCD and in patients with increased risk of impaired mobilization when used in combination with G-CSF [25,26]. The CXCR2 agonist truncated GRO-β (MGTA-145) and the integrin α4β1 inhibitor WU-106 have been explored preclinically in combination with plerixafor for in vivo HSC gene therapy. These G-CSF-free strategies led to rapid mobilization and were found to lead to reduced leukocytosis and IL-6 elevation when compared to settings with G-CSF [21,27,28]. Other mobilization agents which have been evaluated in the context of HSC mobilization include the VLA-4 inhibitor BIO5192, and the small molecule BOP which targets α_9_β_1_/α_4_β_1_ integrins [18,29]. These agents have been shown to have a synergistic effect on mobilization when used in combination with G-CSF and AMD3100 [30]. In addition to novel mobilization agents, strategies such as AI-based mobilization prediction and immune modulation are being explored to address the issue of poor mobilizers [31,32]. In the context of in vivo HSC gene therapy, it is also important to note that transient effects of current mobilization regimens leave a narrow window for adenoviral vector administration, requiring precise timing of vector administration to coincide with the peak of HSC mobilization. On the other hand, mobilization drugs with longer half-life in the circulation might reduce the return of mobilized HSCs to the bone marrow.

#### 2.1.3. Rehoming

Efficient rehoming of transduced HSCs to the bone marrow and their engraftment are critical determinants of sustained therapeutic efficacy of in vivo gene therapy. While mobilization strategies increase access to HSCs, they also disrupt key retention and homing signals which are essential for HSC trafficking [33]. Proteolytic cleavage of adhesion molecules and the loss of chemokine gradients may impair the return of circulating HSCs into the bone marrow niche and their efficient re-engraftment [34,35]. One strategy to improve the rehoming of transduced HSCs to the bone marrow could be to engineer these cells to overexpress the integrin VLA-4 or the chemokine receptor CXCR4, increasing their capacity for adhesion [36,37,38]. In addition, G-CSF mobilization has been shown to transiently remodel the bone marrow microenvironment into a more proteolytic and less retentive state for HSCs. Altered stromal cell function and extracellular matrix composition, proteolytic cleavage of adhesion molecules, disruption of chemokine gradients, and G-CSF–induced bone remodeling collectively impair niche retention signals and reduce the capacity for efficient HSC rehoming [33,39]. Consequently, only a limited fraction of circulating transduced HSCs successfully rehome and contribute to long-term hematopoietic reconstitution. Importantly, in the absence of conditioning, transduced cells must compete with endogenous HSCs for limited niche space, further constraining engraftment efficiency [40]. Within the niche, transduced HSCs may be functionally disadvantaged in competing with resident cells, leading to reduced long-term retention [41]. Additionally, HSCs may preferentially home to other secondary hematopoietic organs, most notably the spleen. This became particularly apparent in our non-human primate (NHP) studies, where the majority of vector genomes were found in the spleen [38,42,43]. We speculate that the splenic reservoir has to be saturated before transduced HSCs traffic to the bone marrow, although notably, as a homing site the spleen is much smaller than the bone marrow.

The information presented here illustrates a central challenge for in vivo HSC gene therapy: strategies increasing the accessibility of HSCs for transduction may simultaneously inhibit their ability to rehome and engraft in the bone marrow. This highlights the need for new approaches that preserve bone marrow niche functions and/or restore critical homing interactions following transduction. Such approaches will require a better understanding of HSC trafficking after mobilization.

### 2.2. Blood

Following intravenous administration, adenoviral vectors intended to target HSCs in the periphery immediately come into close contact with a complex blood environment containing soluble proteins and circulating blood cells. The interactions may trigger immune activation and reduce the fraction of vector available to transduce target cells, representing one of the earliest and most significant barriers to efficient in vivo HSC gene therapy.

#### 2.2.1. Virus Sequestration

**Figure 2:**
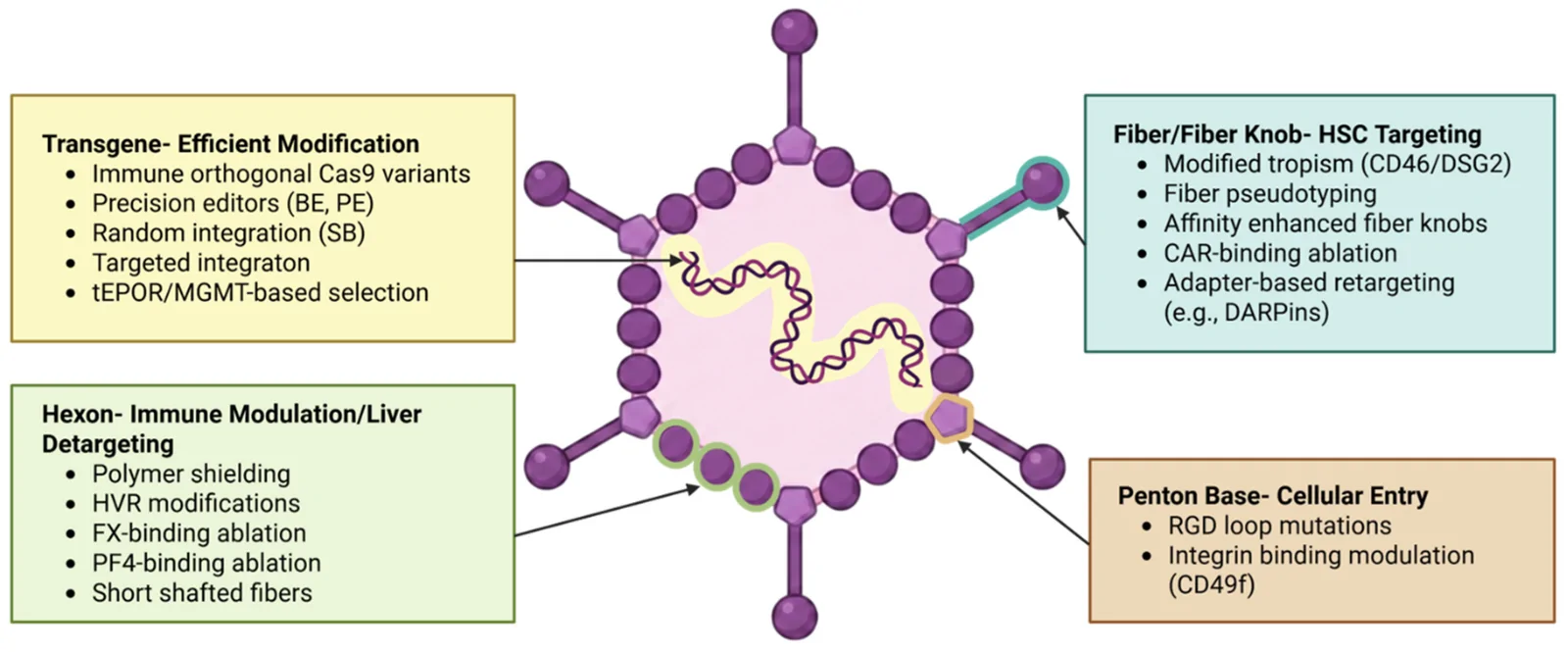
Vector engineering strategies to overcome host barriers. BE, base editor; PE, prime editor; SB, Sleeping Beauty transposase; tEPOR, truncated form of the erythropoietin receptor; MGMT, O6-methylguanine-DNA methyltransferase; CAR, chimeric antigen receptor; DARPins, designed ankyrin repeat proteins; HVR, hypervariable regions; FX, coagulation factor X; PF4, platelet factor 4; RGD, Arginine-Glycine-Aspartic acid. Created in BioRender. Anna K. Anderson. (2026) https://app.biorender.com/illustrations/6a4f12ba1958ad3fbf3e878f.

Soluble factors in the blood play a role in first-generation Ad5 vector sequestration. Adenoviral particles can activate the complement cascade through both antibody (IgM, IgG)-dependent and antibody-independent mechanisms, leading to deposition of complement components such as C3b on the viral capsid [44]. Once adenovirus particles are bound and opsonized by complement proteins they are targeted by immune cells, leading to both vector clearance by phagocytic cells and an inflammatory response [45]. Additionally, coagulation factors, primarily factor X (FX), can interact with the adenovirus hexon proteins or Ad fibers respectively [46,47]. This interaction promotes the targeting of adenovirus to hepatocytes through subsequent binding to heparan sulfate proteoglycan and/or low density lipoprotein receptor-related protein [48]. One strategy to prevent the binding of soluble factors and resultant clearance of virus is the coating of adenoviral particles with polyethylene glycol (PEGylation) to provide a physical barrier against opsonization (Figure 2) [49,50]. Another approach that aims to minimize soluble factor binding employs modified adenovirus capsids with ablated binding sites for coagulation factors [51]. In addition to soluble plasma proteins, circulating blood cells contribute substantially to adenoviral sequestration and immune activation. Carlisle et al. demonstrated that human erythrocytes can bind adenovirus through CAR. They also showed that erythrocytes expressing complement receptor 1 can bind and sequester complement-opsonized viral particles [52]. Innate immune cells further contribute to adenoviral clearance. Antibody bound adenovirus is recognized and phagocytosed by neutrophils, an interaction that can further promote a proinflammatory environment [53]. Platelets bind to adenovirus in the blood, forming aggregates which then accumulate in the liver where they are cleared [54]. Platelet activation leads to the release of proinflammatory cytokines and platelet depletion makes thrombocytopenia a commonly observed side effect of systemic Ad administration [55]. It has recently been suggested that vectors ablated for binding to platelet factor 4 (PF4) may provide a safer alternative [56]. Collectively, these studies based on first-generation adenoviral vectors suggest that interactions between viral vectors and blood cells and proteins substantially reduce the fraction of circulating vector available for HSC transduction while promoting inflammation and thrombotic complications.

#### 2.2.2. Innate Immune Response

The strong inflammatory response associated with systemic adenovirus delivery has been a major concern for adenoviral gene therapies. Of note, an early clinical trial employing a first-generation Ad vector to deliver ornithine transcarbamylase cDNA resulted in the death of a patient from systemic inflammatory response syndrome [57]. Within hours of intravenous administration, viral capsids and/or DNA are recognized by pattern recognition and toll-like receptors on monocytes and macrophages, resulting in a robust, dose dependent, innate immune response. This response is also relevant for HDAd particles, regardless of the fiber protein. In the case of in vivo HSC gene therapy, the innate inflammatory response can be exacerbated by mobilization strategies, which increase the number of proinflammatory cytokine-containing myeloid cells, including macrophages, in the circulation [58].

Detection of viral particles activates multiple innate immune signaling pathways which contribute to the production of proinflammatory cytokines (IL-6, TNFα, IL-1β) and chemokines and to the recruitment of effector cells to clear the virus [59,60,61]. In mild cases, this can lead to flu-like symptoms while in more extreme cases patients can develop cytokine release syndrome (CRS) which has serious and potentially deadly clinical manifestations [62]. The innate immune response remains a barrier for the translation of both traditional and newer generation “gutless” adenoviral gene therapy vectors. To improve the safety profile of systemic adenoviral delivery, strategies have been developed that either suppress host inflammatory pathways or reduce innate immune recognition of the vector itself. Pretreatment with cytokine signaling inhibitors or immunosuppressive drugs has shown to be effective for this purpose. Clinically approved drugs that inhibit cytokine signaling and have shown to be effective in preventing and/or treating CRS include the IL-6 and IL-1 receptor antagonists Tocilizumab and Anakinra [63,64,65]. While cytokine-specific inhibition targets individual inflammatory pathways, broader immunosuppressive approaches have also been explored as a strategy to blunt innate immune activation by limiting macrophage-mediated cytokine production. This includes pretreatment with glucocorticoids such as dexamethasone, which has been shown to reduce inflammatory cytokine release and prevent thrombocytopenia and inflammatory gene induction following first-generation adenoviral administration [66]. A combination of these two strategies has been explored prophylactically to successfully attenuate innate immune responses triggered by HDAd in vivo in rhesus macaques [42] and is currently being used in human trials [5]. In addition to pharmacologic immunosuppression, substantial effort has been directed toward engineering first-generation adenoviral vectors that intrinsically evade innate immune recognition. Polymer shielding strategies employing polyethylene glycol (PEG), N-(2-hydroxypropyl)methacrylamide, or poly(amidoamine) have been shown to reduce innate immune activation and liver vector uptake [67,68]. Building upon passive shielding approaches, more sophisticated “shield and adapter” systems have been developed to simultaneously mask native capsid interactions and redirect viral tropism. These systems typically employ hexon-binding humanized single-chain variable fragments (scFvs) combined with targeting adapters such as designed ankyrin repeat proteins (DARPins) (Figure 2) [69]. Similar technologies could potentially be used to target HSC specific receptors (CD117, CD90, CD34) for in vivo HSC gene therapy. Beyond external shielding strategies, rational capsid engineering has also been employed to genetically modify components of the virion to reduce immunogenicity. The hexon hypervariable regions (HVRs), which can interact with multiple host factors including coagulation proteins and scavenger receptors, have become targets for capsid engineering efforts [46,70]. Modification of the hexon HVRs has been used both to ablate native interactions with host factors, including coagulation protein FX and scavenger receptors, and to introduce reactive sites for the attachment of shielding or targeting moieties, enabling modulation of biodistribution and reduction in innate immune reaction [71,72,73,74]. For example, engineered hexon modifications combined with additional capsid mutations have enabled an intravenously administered first-generation adenovirus platform to achieve targeted transduction of human HSPCs while avoiding innate toxicity [73,74]. While rational capsid engineering focuses primarily on known host interaction sites, broader approaches for adenoviral capsid optimization are also emerging. Early efforts at using directed evolution for adenovirus engineering faced challenges; however, recent advances with the use of peptide insertions to create libraries for screening have led to the development of new protocols that have renewed interest in this approach [75]. Similarly, machine learning-guided capsid design strategies that are being applied successfully to adeno-associated virus (AAV) vectors may provide a useful framework for the development of adenoviral capsids with reduced immunogenicity and improved biodistribution profiles [76]. Together, these approaches highlight the rapid evolution of pharmacologic and engineering strategies aimed at mitigating innate immune responses, a central challenge limiting the safety and clinical translation of systemic adenoviral HSC gene therapy.

#### 2.2.3. Adaptive Immune Response

The host adaptive immune system, including pre-existing and induced humoral immunity, presents another barrier to the efficacy and durability of in vivo adenovirus HSC gene therapy. Much of the human population has been previously exposed to common adenovirus serotypes, particularly Ad5, leading to a high prevalence of pre-existing neutralizing antibodies (NAbs) against these vector serotypes [77]. Additionally, administration of a vector based on a particular adenovirus serotype can induce de novo NAb responses, thereby limiting the feasibility of redosing. Restoration of transduction efficiency following depletion of NAbs highlights their central role as a barrier to efficient in vivo HSC transduction [78]. The use of modified Ad5 vectors, nonhuman Ad serotypes or human Ad serotypes with lower seroprevalence (Ad35, 11, 50, 6, 26, 48, 49) has become an important circumvention strategy [79,80,81,82,83,84,85]. In the context of in vivo HSC gene therapy, Ad6 derived HDAd vectors modified to contain Ad35 or Ad3 fibers have been shown to efficiently transduce HSCs in both mouse and NHPs. Anti-Ad6 NAbs have been detected at relatively low titers in human serum, indicating that these vectors would benefit a larger population of patients [43,83,86]. In addition to humoral immunity, cellular response activation, namely the activation of cytotoxic T-lymphocytes (CTLs), leads to the clearance of transduced cells. In first and second generation adenoviral vectors, viral proteins expressed by transduced cells, including HSCs, are processed into peptides and presented via MHC-I. CD8+ T-cells can then recognize and eliminate these cells [48]. Third generation helper-dependent adenoviral vectors contain no viral coding genes and therefore do not express viral proteins. This feature reduces immunogenicity, but capsid proteins can still be processed and presented by MHC-I, indicating that de novo viral gene expression is not a prerequisite to cellular response activation [87,88]. Additionally, antigen presenting cells (APCs) that take up adenoviral particles process viral proteins and present peptides via MHC-II, leading to detection by CD4+ T-cells and the subsequent activation of B-cells. This promotes the production of Ad-specific NAbs that may persist and limit long term efficacy and redosing [89]. In addition to immune responses directed against adenoviral components themselves, immune recognition of co-delivered gene editing machinery of microbial origin (i.e., Cas9) may limit therapeutic genome editing efficacy [3,4,90]. One potential strategy to overcome this barrier is the identification and use of immune orthogonal Cas9 variants with minimal cross reactivity to pre-existing antibodies, enabling immune evasion and redosing [91]. Aside from the use of alternative Ad serotypes, strategies to mitigate adaptive immune responses include the use of animal-derived adenovirus variants, and screening of patients for preexisting NAbs [92]. Strategies used to reduce innate immune responses such as immunosuppression through the use of broad immunosuppressive agents may also suppress humoral and cellular adaptive immunity. Furthermore, specific immune modulation can be achieved through pre-treatment with antibodies to transiently deplete CD4 T-cells or B-cells, block CD40-CD40 ligand interactions, or block co-stimulation by APCs, as evaluated with first-generation Ad5 vectors in mouse models [93,94,95,96]. Finally, it is important to note that often mouse and NHP models do not fully recapitulate the human immune system and the use of advanced predictive models such as humanized mice and in vitro modelling of immune tissues should be explored for this purpose [97]. Overall, the human adaptive immune response to in vivo HDAd administration poses a significant barrier to safety and efficacy. Nevertheless, continued advances in vector engineering, immune modulation and modeling will continue to strengthen the translatability of this approach.

### 2.3. Tissues

#### 2.3.1. Vector Sequestration

Following the systemic administration of adenoviral vectors, including HDAds, sequestration by the reticuloendothelial system limits vector availability for the transduction of HSCs. For first-generation Ad5 vectors, the liver is the primary site for adenoviral vector sequestration. The vector gets trapped within hepatic sinusoids where liver-resident macrophages (Kupffer cells), rapidly internalize and clear viral particles from circulation [98,99]. Kupffer cell mediated clearance also contributes to a non-linear relationship between vector dose and target-cell transduction. At higher vector doses, Kupffer cells become saturated, resulting in a disproportionate increase in transduction of target cells [84,100]. This complicates the prediction of clinically effective doses, as small animal models with reduced sequestration capacity may not accurately reflect human responses. In addition to Kupffer cell uptake, hepatocytes also contribute significantly to liver sequestration. This process is mediated by vector interactions with coagulation factors (e.g., FX and FIX), complement protein C4BP, and other vitamin K–dependent zymogens [47,51,101,102]. Efforts to reduce hepatic sequestration have focused on both limiting hepatocyte transduction and reducing Kupffer cell-mediated clearance. Modulation of hepatocyte uptake has been achieved through various capsid engineering approaches including the use of short-shafted fiber proteins [103], the introduction of hexon modifications that permit the covalent attachment of shielding molecules [72], strategies designed to reduce blood factor interactions, and targeted mutations within the CAR- and integrin-binding domains of the fiber knob and penton base, respectively [104,105]. HDAd5 or HDAd6-based vectors with Ad3 or Ad6 fiber shafts have greatly reduced uptake by hepatocytes [38,43]. One possible explanation is that these relatively short fiber shafts interfere with the binding of coagulation factors to hexon proteins. In parallel with efforts to limit hepatocyte transduction, strategies have also been developed to reduce Kupffer cell-mediated clearance. Such approaches have included the replacement of the Ad5 hexon HVR with that of Ad6 and the transient depletion of Kupffer cells prior to vector administration [106,107]. Collectively, these efforts highlight the importance of reducing hepatic sequestration to both minimize toxicity and improve vector availability for efficient HSC transduction. For in vivo HSC gene therapy, these strategies to further reduce liver tropism of HDAds still have to be implemented.

#### 2.3.2. Biodistribution

Preclinical studies using reporter-based, first-generation Ad5 vectors have demonstrated broad dissemination following intravenous delivery, with significant accumulation in organs such as the liver, spleen, lungs, and kidneys [108,109]. This widespread distribution, coupled with uptake by reticuloendothelial cells, reduces the effective concentration of vector available for transduction of target cells. Biodistribution patterns are shaped in part by the receptor tropism of the adenoviral vectors. Strategies for in vivo HSC transduction have moved away from the use of vectors targeting the widely expressed CAR. They instead utilize capsid-modified vectors containing fibers from group B adenoviruses, namely Ad35, which target CD46, a complement regulatory protein expressed on human hematopoietic cells [110,111]. CD46 is expressed at higher levels on primitive HSC than on more differentiated blood cells and tissues. A certain CD46 threshold density seems to be required for Ad infection [112], which could be explained by the fact that one Ad particle needs to bind to several CD46 receptors in close proximity to trigger receptor clustering and signaling involved in integrin-mediated uptake. We speculate that this CD46 receptor threshold is not reached on most non-hematopoietic cells, which explains the preferential targeting of HSCs with higher CD46 expression. More recently, an alternative targeting strategy has emerged using vectors incorporating Ad3-derived fibers which bind desmoglein-2 (DSG2), a junctional adhesion molecule expressed on epithelial and certain hematopoietic cell populations [38,43]. These receptor-targeting approaches have enabled improved transduction of HSCs and modulation of vector tropism; however, receptor-expressing cells in non-target tissues can still be transduced. In this context, it is notable that DSG2 in epithelial cells is expressed in lateral junctions and is not accessible to intravenously injected DSG2-targeting Ads. Off-target transduction has important safety implications as unintended transgene expression in non-hematopoietic lineages may have associated toxicity. One potential strategy to overcome this challenge is the use of cell type specific promoters to express transgenes only in specific HSC-derived lineages (e.g., erythroid lineage cells using large transcriptional control regions [113]).

Moving forward, the accurate assessment of vector biodistribution for safe clinical translation depends on appropriate preclinical models which mimic the receptor expression patterns found in humans [96,114]. Conventional mouse models do not express CD46 or DSG2 at levels or in a pattern characteristic of human biology, leading to the development of transgenic mouse models which better recapitulate human expression patterns for these receptors [115,116]. Despite this, transgenic mouse models for the study of biodistribution have limitations resulting from species specific differences in metabolism, anatomy and physiology. Importantly, DSG2 transgenic mice do not express DSG2 in hematopoietic cells. NHP models more closely reflect the receptor expression and systemic distribution patterns found in humans, providing a more physiologically relevant system for in vivo HSC gene therapy studies. The use of these refined model systems to better predict human biodistribution, alongside vector engineering strategies to target HSCs and limit transgene expression to desired cell populations, provides a path towards the development of safer and more effective in vivo HSC gene therapies.

#### 2.3.3. Liver Toxicity

When first-generation adenoviruses are sequestered in the liver, clearance of Ad-transduced hepatocytes and activation of innate immune components leads to liver injury, characterized by elevated aminotransferase levels (AST, ALT) and necrosis. Hepatocyte transduction can occur independently of CAR binding [47,117], and hepatotoxicity has been attributed to both innate and adaptive immune responses: Kupffer cell uptake triggers inflammatory chemokine [118,119,120] and cytokine release that recruits neutrophils, NK cells, and γδ T cells to the liver, exacerbating inflammation and tissue damage [121,122,123], while virus-specific cytotoxic T-lymphocytes kill transduced hepatocytes in an IFN-γ-dependent manner, causing widespread necrosis [124,125]. Accordingly, strategies that reduce hepatic sequestration (Section 2.3.1) have consistently lowered inflammatory cytokine release, hepatocyte transduction and hepatotoxicity severity. In HDAd-mediated in vivo HSC gene therapy, for example, HDAd6/35 and HDAd6/3 vectors—which contain hexon and penton domains from Ad6 and fibers targeting HSC-enriched receptors—reduce liver cell transduction and inflammatory cytokine release [43,83].

### 2.4. HSCs

#### 2.4.1. HSC Rarity and Targeting

True long-term HSCs (LT-HSC) with self-renewal capacity comprise only about 1 in 10,000 to 1 in 100,000 cells in the bone marrow. Targeting this rare cell population poses a significant challenge and requires the engineering of adenovirus capsid proteins to interact with specific cellular receptors expressed on HSCs. There are over 50 Ad serotypes, grouped A-G, most of which use CAR, a receptor expressed only at very low levels in HSCs, as their primary attachment receptor. Group B viruses, however, can attach to cells via the CD46 or DSG2 receptors, both of which are expressed on HSCs [73]. Ad serotypes 3, 11 and 35 are known to interact with the CD46 receptor while Ads 3,7,11 and 14 can bind cells through DSG2 [126,127]. Several groups have achieved efficient transduction of CD34+ cells using Ad5 or Ad6 derived vectors containing Ad35 fibers targeting CD46 [20,83,128,129,130]. This system has been improved using libraries to identify mutations in the Ad35 fiber knob which increase its affinity for CD46 [20,131]. Similarly, Ad5 or Ad6 derived vectors have been modified to target DSG2 expressing cells through Ad14 or affinity enhanced Ad3 fiber knobs [38,73,86]. Notably, DSG2 is not expressed on mature erythrocytes, leading to reduced sequestration of DSG2-targeting vectors by these cells, which is overwhelmingly dominant in blood [73]. These vectors have also been found to target more primitive HSC populations than vectors targeting CD46, consistent with the finding that CD46 is expressed on all hematopoietic cells while DSG2 expression is restricted to an HSC-enriched population [38,73]. While Ad35 and Ad3 fiber-based approaches have significantly improved HSC targeting, other flexible adenovirus targeting approaches, developed for first generation vectors, exist and could be applied to enable specific transduction of HSCs by HDAd vectors. One potential novel strategy is the use of adenovirus-antibody site-specific covalent conjugates which retarget viral particles via antibodies covalently linked to engineered sites on capsid proteins, such as the fiber knob [132,133]. While promising, the manufacturing of these conjugates at large scale could be complex and costly. Other approaches are adapter-based, and include the use of aforementioned DARPins to redirect the virus by binding both it and the target cell receptor with a high level of specificity [134,135,136]. The direct insertion of CD34, CD90, or CD117 ligands into the Ad fiber or penton seems to be problematic due to the complex structure of Ad capsid proteins. Collectively, these approaches highlight that the extreme rarity of LT-HSCs necessitates highly efficient and specific targeting, with capsid engineering currently representing the primary strategy for directing adenoviral vectors to these cells, while emerging modular retargeting platforms offer additional flexibility and could potentially be optimized for HSC-specific delivery.

#### 2.4.2. Cellular Uptake, Endosomal Escape and Nuclear Transport

Following primary receptor binding, the adenoviral particle must be internalized and released from the endosome formed during this process. In order for gene therapies to be effective, the vector must then deliver its DNA to the host cell nucleus where it can be transcribed. Adenoviruses have evolved mechanisms to carry out these processes efficiently. This said, inefficiencies at certain steps of this process, specifically for the transduction of HSCs, present barriers to successful in vivo therapies. The internalization of adenovirus by the cell is mediated by the interaction of the viral penton protein with cellular integrins. For most human adenovirus serotypes, this involves the binding of penton base RGD amino acid loops to *α*_v_ integrins (α_v_β_3_ and α_v_β_5_) on the cell surface [137]. *α*_v_ integrins are expressed at low levels on HSCs, and widely on differentiated cell types, leading to suboptimal internalization in HSCs and interactions with non-target cells [138]. In particular, the interaction that takes place between macrophage β_3_ integrins and penton base RGD loops contributes to the innate immune response by facilitating the activation of proinflammatory pathways [139]. Recently, researchers have sought to overcome these issues by replacing HVR RGD loops with a sequence from human laminin subunit α1, which interacts with α6 integrins (CD49f) [140]. CD49f is widely expressed on stem cells, making it an ideal target to promote the selective transduction of HSCs [141]. Using DSG2-targeting Ad5/3 vectors containing this modification, increased targeting selectivity for LT-HSCs and reduced activation of inflammatory cytokine have been reported [73,74,86]. The strategy to target the Ad simultaneously to the high affinity attachment (DSG2) and an alternative uptake receptor (CD49f) (both of which are expressed on primitive HSCs) could therefore be considered for HDAds to be used for stable in vivo HSC transduction. In the next step of the viral uptake process, integrin binding triggers the clathrin-dependent endocytosis or macropinocytosis of the viral particle, depending on the adenoviral species [142,143]. Once inside the cell, the Ad5 particle is rapidly released from the endosome and undergoes stepwise degradation before it travels along structural proteins to the nucleus to deliver its DNA, directed by nuclear localization signals on viral proteins. Intracellular vector trafficking pathways inside the cell vary and can be influenced by the entry receptor used, as shown with Ad5/35 vectors [144]. In some pathways, retention of viral particles within endosomes and recycling of these endosomes to the cell surface can lead to reduced levels of gene transfer. These findings demonstrate that while in vivo HSC gene therapy takes advantage of the efficient mechanisms utilized by adenoviruses for intracellular trafficking, viral uptake can be further optimized to improve the specific transduction of HSCs. Endosomal escape and intracellular trafficking therefore remain important targets for further optimization.

#### 2.4.3. Quiescence

Stable genetic modification of LT-HSCs is required to achieve durable therapeutic efficacy of in vivo gene therapy. The majority of LT-HSCs exist in a nondividing quiescent state, remaining in the G0 cell cycle phase. This is problematic for the use of gene editing strategies that rely on homology directed repair (HDR) which is restricted to the G2/S phases. Gene editing is achieved through the use of programmable site-specific endonucleases which introduce double strand breaks (DSBs) to pre-determined genomic sites. Cellular repair of DSBs primarily occurs through either non-homologous end joining (NHEJ) or HDR. NHEJ is inherently error-prone and is typically exploited for gene disruption or excision, whereas HDR enables precise gene correction or insertion using a donor template. NHEJ is the favored pathway in quiescent HSCs, limiting the utility of HDR for the in vivo modification of these cells. Importantly, several gene editing strategies that do not rely on HDR have emerged and are well-suited for modifying quiescent HSCs for the treatment of various diseases and inherited disorders [145]. The high payload capacity (over 30 kb) of new generation adenoviral vectors gives them the ability to deliver any of these gene editing technologies. For HDR independent, targeted gene integration, the homology-independent targeted integration (HITI) or precise integration into target chromosome (PITCh) systems have demonstrated utility. These strategies rely on NHEJ and microhomology-mediated end-joining (MMEJ) respectively and are not restricted to any particular cell cycle phase [146,147,148]. For random transgene integration, the Sleeping Beauty (SB) transposase system has successfully conferred stable gene transfer into HSCs [2,149,150]. Additionally, base and prime editors are independent of creating DSBs. These approaches introduce single-strand nicks and leverage endogenous DNA mismatch repair pathways to achieve precise sequence modification. Base editors delivered via helper-dependent adenoviral vectors have been effectively utilized to introduce precise nucleotide substitutions to HSCs in vivo in mouse models [4,151]. Prime editors, which can be engineered to introduce various types of insertions, deletions and substitutions, have also demonstrated potential for efficient in vivo HSC modification [3]. Building upon this, the recently developed PASSIGE system uses prime editing technology to install specific landing sites for recombinases to catalyze the insertion of large DNA elements; this allows for the targeted insertion of large genes while avoiding both DSBs and HDR [152]. Notably, HDR-mediated editing can still yield robust functional outcomes in vivo, particularly when coupled with selective expansion of edited cells; however, the intrinsic efficiency of HDR within quiescent LT-HSCs likely remains limited [153]. Collectively, these advances highlight that while HSC quiescence remains a fundamental biological constraint, emerging editing strategies are increasingly able to circumvent cell cycle limitations to enable efficient and durable precision genome modification.

#### 2.4.4. Competition

For in vivo HSC gene therapies to achieve long-term therapeutic benefit, modified HSCs must persist and contribute to hematopoiesis. This presents a challenge, as unlike ex vivo transplantation protocols employing myeloablative conditioning, most in vivo approaches leave the endogenous HSC compartment intact, requiring modified cells to compete directly with resident LT-HSCs for occupancy of the bone marrow niche. HSC fitness, self-renewal capacity, or proliferative potential can be impaired by adenovirus transduction, genome editing or transgene expression. This can lead to the slow loss of the gene modified cell population as these cells are outcompeted by the unmodified HSCs [42]. Competition becomes an even greater obstacle when therapeutic efficacy requires modification of a large proportion of the HSC compartment [4]. In such cases, editing levels may not be sufficient and enrichment of the modified HSC population may be required. This barrier has led to the exploration of strategies to enrich modified HSCs, either through transient selection or by conferring a selective advantage to modified cells. One mechanism for selection is the expression of an O6-methylguanine-DNA methyltransferase mutant (i.e., MGMT^P140K^) in modified cells, combined with chemoselection using O6-Benzylguanine (O^6^BG) and an O^6^BG alkylating agent (e.g., BCNU/Carmustine, temozolomide). MGMT^P140K^ protects transduced cells from the chemotherapeutic agents, allowing for their enrichment by the selective killing of unmodified cells [154,155]. This system has been utilized for in vivo HSC gene therapy using HDAd vectors, resulting in stable gene expression in up to 80% of peripheral blood cells [18,156]. Recently, efforts have been made to uncover new MGMT mutants which can be generated via base editing in order to improve this system by eliminating the need for transgene insertion [157]. While effective, the requirement for chemotherapy may limit the applicability of this approach, particularly for diseases in which treatment-related toxicity cannot be readily justified.

A number of groups are working on CD45- and CD117-directed genome editing strategies to shield engineered HSCs from corresponding antibody–drug conjugates, CAR T-cells or function-depleting monoclonal antibodies, which are capable of eliminating untransduced HSCs [158,159,160]. These elegant strategies bear however the risk of unspecific toxicity of the depleting agents.

An alternative strategy is to selectively expand modified HSCs rather than selectively deplete unmodified cells. One such approach is the expression of a truncated form of the erythropoietin receptor (tEPOR). tEPOR lacks the negative regulatory domain of the receptor, increasing responsiveness to endogenous erythropoietin and promoting preferential expansion of modified erythroid progenitors [161]. This approach shows particular promise for the treatment of hemoglobinopathies where anemia contributes to elevated levels of endogenous EPO which are typically sufficient for the selective expansion of tEPOR expressing HSCs [162,163].

Vaccine-mediated expansion of engineered B-cells is another approach to increase the blood concentration of broadly neutralizing antibodies and other secreted proteins. B-cells, the precursor of plasma cells expressing a specific B-cell receptor (BCR), i.e., a membrane-bound Ig, expand after immunization with the antibody’s cognate antigen, mostly in lymph nodes. A common BCR engineering approach has been to insert a cassette comprising both a complete light chain and a variable heavy chain (VH) domain into the IgH locus, at locations between the VDJ region and the alternate constant region genes [164,165,166,167]. A consequence of this approach is that the Fc domain of the resulting BCR and antibody is provided by endogenous sequences, with the specific isotype determined by the natural process of class switch recombination.

Any strategy that confers a proliferative advantage to modified HSCs must also consider the potential risk of clonal expansion and malignant transformation. This risk can be managed using the careful selection of promoters that keep expression at paraphysiological levels, the co-expression of suicide genes, or inducible expression systems [168,169,170]. Continued development of safe, controllable methods for selective enrichment of modified cells will likely be essential for achieving consistent long-term therapeutic benefit without the need for myeloablative conditioning.

## 3. Conclusions

Over the past several decades, HDAd technology has advanced significantly, enabling its application to expand to in vivo HSC gene therapy. In parallel, in vivo HSC gene therapy has progressed from proof-of-concept studies to early-phase clinical translation. As the field has advanced, the remaining limitations have shifted from vector development toward the interactions between the vector and host. This review has examined the major host barriers that constrain the safety and efficacy of in vivo HDAd-mediated HSC gene therapies. The localization of HSCs within the difficult-to-access bone marrow niche presents unique challenges related to the mobilization and rehoming of these cells. Following systemic administration, HDAd vectors encounter soluble factors and blood cells, leading to sequestration as well as innate and adaptive immune responses. They are also widely distributed throughout the body, where interactions with off-target tissues, particularly the liver, can result in organ toxicities while reducing the vector available for HSC transduction. Finally, HSCs are rare, predominantly quiescent in vivo, and must compete with endogenous cells following transduction, making the stable modification of a large fraction of the HSC pool difficult. Taken together, these challenges highlight that the continued success of in vivo HSC gene therapy depends on a detailed understanding of host barriers and the development of engineering strategies to overcome them.

Importantly, recent advances suggest that many of the challenges described here are not fixed biological limitations but can be addressed through continued engineering and therapeutic innovation. Improved mobilization and rehoming strategies will enhance access to niche-resident HSCs, while capsid engineering and shielding approaches will improve HSC targeting, reduce off-target uptake, and minimize organ toxicities. These same vector engineering strategies can also reduce immune recognition and may be complemented by immunomodulatory protocols to further improve safety and efficacy. At the level of the HSC, advances in genome editing technologies and selective expansion systems are expected to increase both the efficiency and durability of genetic modification. Ultimately, progress toward widely applicable HDAd-mediated in vivo HSC gene therapy will likely depend not on a single technological breakthrough, but on the integration of advances that improve access to the bone marrow niche, reduce vector clearance and off-target uptake, enhance HSC-specific delivery, enable efficient genome modification, and promote the long-term persistence of genetically modified HSCs.

The initiation of clinical trials employing HDAds provides encouraging evidence that the strategies discussed here are overcoming the biological barriers that have historically limited the translation of in vivo HSC gene therapy. An ongoing Phase 1/2 clinical trial is using an HDAd platform via intravenous vector delivery with immune prophylaxis to transduce HSCs in vivo for the treatment of X-linked chronic granulomatous disease (X-CGD) in certain adult male patients [5]. Beyond evaluating therapeutic efficacy, this study will provide valuable insights into vector-host interactions, including vector biodistribution, immune responses, and the long-term persistence of genetically modified HSCs. These findings will not only inform the continued optimization of HDAd vectors for HSC gene therapy but also advance the development of the platform more broadly. Notably, β-hemoglobinopathies and HIV are particularly attractive targets for in vivo HSC gene therapy, and encouraging preclinical results using HDAd vectors demonstrate the potential for future clinical translation [3,4,43,171]. For these diseases in particular, eliminating the need for ex vivo transplantation and myeloablative conditioning could help lower one barrier to curative therapies, especially in low- and middle-income countries where disease burden is greatest.

The versatility of HDAd vectors positions them to expand the scope of in vivo gene therapy beyond the applications currently under investigation. Their large payload capacity enables delivery of complex gene editing systems, while modular capsids facilitate flexible engineering and retargeting, and scalable manufacturing supports clinical translation. Emerging applications include the in vivo generation of engineered immune cells, such as CAR-T cells, highlighting the potential of HDAd platforms to extend beyond conventional HSC gene replacement. Rather than representing fixed biological barriers, the host-vector interactions discussed throughout this review provide a framework for the continued rational engineering of helper-dependent adenoviral vectors. Together, these advances have the potential to establish HDAds as a safe, durable, and broadly applicable platform for in vivo HSC gene therapy.

## Data Availability

No new data were created or analyzed in this study. Data sharing is not applicable to this article.

## Abbreviations

The following abbreviations are used in this manuscript:

AAV	adeno-associated virus
Abs	antibodies
Ad	adenovirus
ALT	alanine aminotransferase
APCs	antigen presenting cells
AST	aspartate aminotransferase
BCR	B-cell receptor
BE	base editor
BM	bone marrow
CAR	coxsackie and adenovirus receptor
CRS	cytokine release syndrome
CTLs	cytotoxic T-lymphocytes
CXCL12	C-X-C motif chemokine ligand 12
CXCR-4	C-X-C chemokine receptor type 4
DARPins	designed ankyrin repeat proteins
DSBs	double strand breaks
DSG2	desmoglein-2
FDA	food and drug administration
FX	factor X
G-CSF	granulocyte colony stimulating factor
GRO-β	Growth-Regulated Oncogene-beta
HDAd	helper-dependent adenovirus
HDR	homology directed repair
HER2	Human Epidermal Growth Factor Receptor 2
HITI	homology-independent targeted integration
HSC	hematopoietic stem cell
HSPCs	hematopoietic stem cell and progenitors
HVR	hypervariable regions
IFN-γ	interferon gamma
IgM, IgG	immunoglobulin M, immunoglobulin G
IL-6	interleukin 6
LT-HSC	long-term HSCs
MGMT	O6-methylguanine-DNA methyltransferase
MHC-I	major histocompatibility complex class I
MMEJ	microhomology-mediated end-joining
NAbs	neutralizing antibodies
NHEJ	non-homologous end joining
NHP	non-human primate
NK cells	natural killer cells
PASSIGE	prime-editing-assisted site-specific integrase gene editing
PE	prime editor
PEG	polyethylene glycol
PF4	platelet factor 4
PITCh	precise integration into target chromosome
RGD	Arginine-Glycine-Aspartic acid
SB	Sleeping Beauty transposase
SCD	sickle cell disease
tEPOR	truncated form of the erythropoietin receptor
TNFα	tumor necrosis factor alpha
VCAM-1	vascular cell adhesion molecule 1
VLA-4	Very Late Antigen-4
X-CGD	X-linked chronic granulomatous disease
